# In Vitro and In Vivo Biocompatibility of Natural and Synthetic *Pseudomonas aeruginosa* Pyomelanin for Potential Biomedical Applications

**DOI:** 10.3390/ijms24097846

**Published:** 2023-04-25

**Authors:** Mateusz M. Urbaniak, Małgorzata Gazińska, Karolina Rudnicka, Przemysław Płociński, Monika Nowak, Magdalena Chmiela

**Affiliations:** 1 Department of Immunology and Infectious Biology, Faculty of Biology and Environmental Protection, University of Łódź, 90-237 Łódź, Poland; 2 The Bio-Med-Chem Doctoral School, University of Lodz and Lodz Institutes of the Polish Academy of Sciences, 90-237 Łódź, Poland; 3 Department of Engineering and Technology of Polymers, Faculty of Chemistry, Wrocław University of Science and Technology (WUST), 50-370 Wrocław, Poland

**Keywords:** pyomelanin, *Pseudomonas aeruginosa*, chemical structure, biocompatibility

## Abstract

Bacteria are the source of many bioactive compounds, including polymers with various physiological functions and the potential for medical applications. Pyomelanin from *Pseudomonas aeruginosa*, a nonfermenting Gram-negative bacterium, is a black–brown negatively charged extracellular polymer of homogentisic acid produced during L-tyrosine catabolism. Due to its chemical properties and the presence of active functional groups, pyomelanin is a candidate for the development of new antioxidant, antimicrobial and immunomodulatory formulations. This work aimed to obtain bacterial water-soluble (Pyo_sol_), water-insoluble (Pyo_insol_) and synthetic (sPyo) pyomelanin variants and characterize their chemical structure, thermosensitivity and biosafety in vitro and in vivo (*Galleria mallonella*). FTIR analysis showed that aromatic ring connections in the polymer chains were dominant in Pyo_sol_ and sPyo, whereas Pyo_insol_ had fewer C_ar_-C_ar_ links between rings. The differences in chemical structure influence the solubility of various forms of pyomelanins, their thermal stability and biological activity. Pyo_sol_ and Pyo_insol_ showed higher biological safety than sPyo. The obtained results qualify Pyo_sol_ and Pyo_insol_ for evaluation of their antimicrobial, immunomodulatory and proregenerative activities.

## 1. Introduction

*Pseudomonas aeruginosa*, a nonfermenting Gram-negative bacterium that is widespread in the environment and is an opportunistic pathogen, produces a variety of pigments, including pyocyanin, pyorubin, pyoverdine and pyomelanin. Pyomelanin is a negatively charged extracellular polymer of homogentisic acid (HGA) that is produced during L-tyrosine catabolism [1]. L-tyrosine is converted to 4-hydroxyphenylopyruvic acid (4-HPPA) by tyrosine aminotransferase, and then 4-HPPA is biotransformed to HGA by 4-hydroxyphenylpyruvate dioxygenase. Pyomelanin then forms spontaneously when secreted HGA autoxidizes to benzoquinone acetic acid, which then undergoes polymerization to form pyomelanin chains. Similar to chemically synthesized polymers of HGA, pyomelanin is a dark brown to black pigment [2,3]. The chemical structure of melanins, including pyomelanin, determines their oxidizing (quinone groups) and reducing (hydroxyquinone groups) properties that allow them to accept or donate electrons [4,5].

The primary function of microbial melanins is to protect cells from UV radiation [6]. In addition, pyomelanin increases the effectiveness of bacterial adhesion to surfaces, thus supporting the formation of biofilms and the extracellular transfer of electrons [7]. The production of pyomelanin by bacteria in the marine bacterial genus *Pseudoalteromonas* is induced during biofilm formation and under heat stress, suggesting that this pigment is involved in the adaptation of these bacteria to grow in a hostile ecological niche [8]. Pyomelanin is produced by clinical *P. aeruginosa* isolates from patients with chronic lung infections. It remains unclear whether pyomelanin is relevant to the pathogenicity of these bacteria; its production is linked to a reduction in oxidative stress, which may be an important trait to improve bacterial survival within pulmonary macrophages [9,10]. Additionally, melanins of *Aspergillus fumigatus* protect the fungus from host reactive oxygen intermediates [11]. A study by Fonseca et al. revealed that metal binding and metal reduction by pyomelanin are necessary for iron acquisition [7]. Due to the antimicrobial and antioxidant activities of microbial melanins, including pyomelanin produced by *P. aeruginosa,* they can be used in the food industry to coat packaging to prolong food expiration dates [5]. Bacterial melanins can be exploited as dyes and colorants and used in cosmetics as sunscreen and oxidative stress-reducing substances, whereas in agriculture, they may help to retain metals and improve iron availability in the soil [12]. These compounds are promising candidates for the design of new drugs, including antitumor drugs, vaccine antigen carriers, adjuvants and optoacoustic imaging contrasts [13,14,15]. The treatment of infections with pyomelanin with an accompanying inflammatory reaction driven by oxidative stress has also been considered [16]. The increased sensitivity of several bacterial pathogens to antibiotics in the milieu of pyomelanin has also been demonstrated, as well as the antimicrobial activity of melanin itself and its iron ion complexes against *Helicobacter pylori*, *Candida albicans* and human immunodeficiency virus (HIV) [17,18].

Eukaryotic and bacterial melanins may exhibit immunomodulatory properties. Cuttlefish melanin is able to activate and polarize dendritic cells, while human neuromelanin activates the nuclear factor kappa B (NF-κB) signaling pathway resulting with the secretion of pro-inflammatory cytokines: tumor necrosis factor alpha (TNF-α) and interleukin (IL)-6 [19]. The role of melanin in the regulation of innate immunity in humans is potentially due to the modulation of phagocytes and complement activity, reduction in the mRNA translation of proinflammatory cytokines and interaction with effector molecules [20]. Melanization is an important part of the cuticular wound healing process in arthropods and functions as part of the innate immune system in isopods by encapsulating parasites with melanin [21]. However, negatively charged melanin isolated from *Cryptococcus neoformans* diminished fungal cell susceptibility to cationic antimicrobial peptides of phagocytes, which was correlated with the downregulation of phagocytosis and diminished secretion of TNF-α, IL-1β, IL-6 and IL-12 as well as modulation of complement activities [22]. The potential wide range of microbial melanin applications drives studies on natural, synthetic and recombinant melanins [23].

Hypothetically, natural bacterial or synthetic formulations of pyomelanin may vary in their biological activity. Before studying different applications of pyomelanin formulations, it seems valuable to verify whether there are differences in physicochemical properties, cytocompatibility, the ability to stimulate the activation of the NF-κB pathway and in vivo toxicity between bacterial pyomelanin and its synthetic form.

Considering the potential application of pyomelanin in medicine, the aim of this study was to characterize the biochemical and physiological properties of natural water-soluble and water insoluble pyomelanin (Pyo_sol_ and Pyo_insol_) isolated from a culture of *P. aeruginosa* and synthetic pyomelanin (sPyo), obtained by us under laboratory conditions, for further biomedical applications. We optimized *P. aeruginosa* growth conditions for the effective production of pyomelanin, developed an isolation procedure for natural water-soluble pyomelanin and a procedure for pyomelanin synthesis. The chemical structure of the bacterial and synthetic pyomelanin was investigated by Fourier transform infrared (FTIR) spectroscopy and the thermal properties were characterized by means of differential scanning calorimetry (DSC) and thermogravimetry (TGA). The cytocompatibility of pyomelanin from both sources was determined using an in vitro model on reference L-929 fibroblasts and THP-1 human monocytes. Noncytotoxic concentrations were established in the reference MTT reduction assay. In our in vivo biosafety study, we used the *Galleria melonella* insect model as an alternative to rodents. Larvae *of G. mellonella* are widely used in the toxicity assessment of new biopharmaceuticals, bioactive substances and newly synthesized chemicals [24]. Due to the ability to maintain larvae in a temperature range from 20 °C to 42 °C, this insect model mimics the physiological conditions of mammals during in vivo toxicity assessment [25]. In addition, *G. melonella* larvae provide an inexpensive and convenient way, free from legal or ethical restrictions, to assess the safety of new biomolecules, and the results generated from this model show a strong correlation with those obtained from mammalian systems. The use of *G. mellonella* larvae provides a more accurate representation of the biocompound’s interactions with the host organism than studies on cell lines, which allows for the precise selection of concentrations before animal testing [26]. Furthermore, the ability of pyomelanin to activate the nuclear factor kappa B (NF-κB) signaling pathway was determined using the human recombinant monocyte model THP1-Blue™ NF-κB.

## 2. Results

### 2.1. The Efficiency of the Pyomelanin Biosynthesis and Chemical Synthesis

To determine the application potential of different pyomelanin variants, we first analyzed the extraction and synthesis efficiencies. We extracted two variants of natural pyomelanin produced by *P. aeruginosa* during growth on pyomelanin minimal medium (PMM) dedicated to these bacteria: PMM I, PMM II and pyomelanin synthesized from HGA. The efficiency of the extraction of natural pyomelanin formulations vs. the efficiency of pyomelanin synthesis is shown in Table 1. Both variants of natural pyomelanin, Pyo_insol_ and Pyo_sol_, were produced more efficiently by *P. aeruginosa* after 7 days of growth of this bacterium on PMM II than by PMM I medium (1.79 ± 0.18 g/L and 1.22 ± 0.10, respectively, *p* < 0.05) (Table 1). Compared to the high-efficiency bacterial biosynthesis of pyomelanin, the yield of sPyo production from HGA was equal to 0.077 ± 0.01 g/1 g HGA.

### 2.2. Identification of the Functional Groups and Linkages in Pyomelanin Molecules

**Figure 1:**
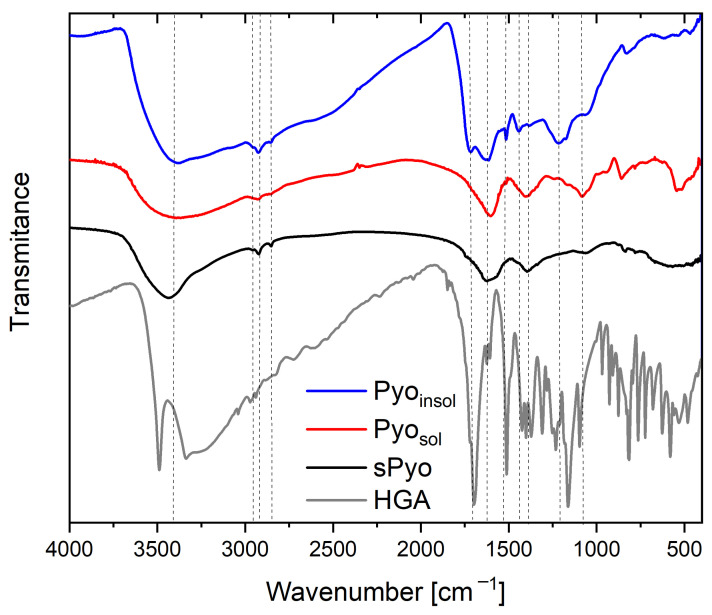
Fourier transform infrared (FTIR) spectra of the water-soluble pyomelanin (Pyo_sol_), water-insoluble pyomelanin (Pyo_insol_), synthetic pyomelanin (sPyo) and homogentisic acid (HGA).

The structure of the pigments was investigated by FTIR spectroscopy, a technique in which a sample’s absorbance of infrared light at various wavelengths is measured to determine the structure of molecules. The infrared spectrum includes absorbance bands corresponding with the various vibrations of the sample’s atoms. Each chemical molecule will produce a unique infrared spectrum. It should be mentioned that structural characterization of melanin is elusive due to its complexity and metabolic residues, such as proteins, amino acids and carbohydrates, in cases of microbial origin. The FTIR spectra of microbial Pyo_sol_ and Pyo_insol_ and synthetic sPyo are presented in Figure 1. The FTIR spectra of bacterial Pyo_sol_ and Pyo_insol_ were similar at wavenumbers higher than 1600 cm^−1^. Between 3700 and 3000 cm^−1^, a large absorption band resulted from overlapping -OH groups and unsaturated carbon or aromatic rings.

At lower wavenumbers, the bands with three maxima assigned to the stretching vibrations of aliphatic C–H were at 2953, 2925, and 2855 cm^−1^ in Pyo_insol_ and 2960, 2944, and 2873 cm^−1^ in Pyo_sol_. The band at 1700 cm^−1^ corresponding to carbonyl stretching (C=O) of the COOH groups was visible in Pyo_insol_, whereas this band was absent in Pyo_sol_. The bands at 1631 and 1613 cm^−1^ in Pyo_insol_ and at 1601 cm^−1^ in Pyo_sol_ were typical for C=C bonds conjugated with C=O groups (quinones). At wavelengths shorter than 1600 cm^−1^, the differences between the spectra of Pyo_sol_ and Pyo_insol_ were more considerable. The bands at 1515 cm^−1^ ascribed to aromatic C_ar_–H bonds, and at 1441 cm^−1^ from in-plane aromatic skeletal vibrations of C=C, were only visible in Pyo_insol_. Similarly, the band of the phenolic–OH links at 1216 cm^−1^ appeared only in the Pyo_insol_ FTIR spectrum. The band associated with the O–H bonds of hydroxyl groups attached to the ring was strong at 1402 cm^−1^ in Pyo_sol_ and weak at 1385 cm^−1^ in Pyo_insol_. The strong band at 1082 cm^−1^ in Pyo_sol_ and the shoulder band at 1065 cm^−1^ in Pyo_insol_ could be related to the stretching of a C–O band of a primary alcohol group (phenolic groups). The strong band at 857 cm^−1^ in Pyo_sol_ and weak band at 828 cm^−1^ in Pyo_insol_ were related to out-of-plane deformation vibrations of aromatic C_ar_–H bonds.

The FTIR spectrum of sPyo in the range of 4000–1600 cm^−1^ was similar to the spectra of both types of bacterial pyomelanin, except that the band assigned to the O–H stretch had a narrower range of 3700–3300 cm^−1^. The bands corresponding to aliphatic C–H bonds were at 2957, 2924 and 2852 cm^−1^. The sPyo spectrum also presented a weak shoulder peak at 1738 cm^−1^ from COOH groups. The bands at 1515 and 1216 cm^−1^ visible for Pyo_insol_ were absent, similar to that of Pyo_sol_. There were bands at 1395 and 1062 cm^−1^ from O–H and C_ar_–O vibrations of hydroxyl groups attached to the ring, and bands from aromatic C–H bonds at 835 and 780 cm^−1^. In the polymer chain of sPyo, there were more C_ar_–C_ar_ linkages between rings, as indicated by the absence of a band at 1515 cm^−1^.

### 2.3. The Thermal Stability and Thermal Properties of Pyomelanins

**Figure 2:**
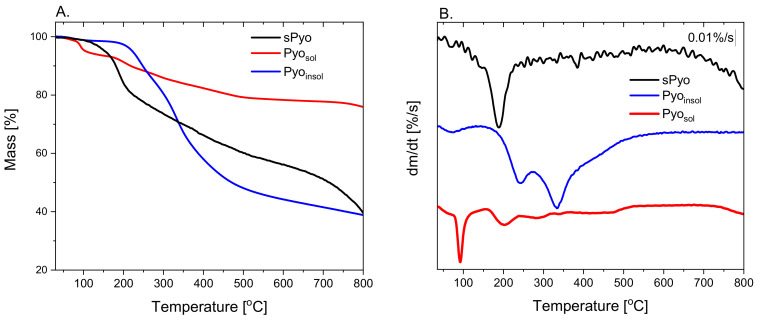
The thermogravimetry (TGA) (**A**) and the first derivative dm/dt (**B**) curves of the pyomelanins.

**Figure 3:**
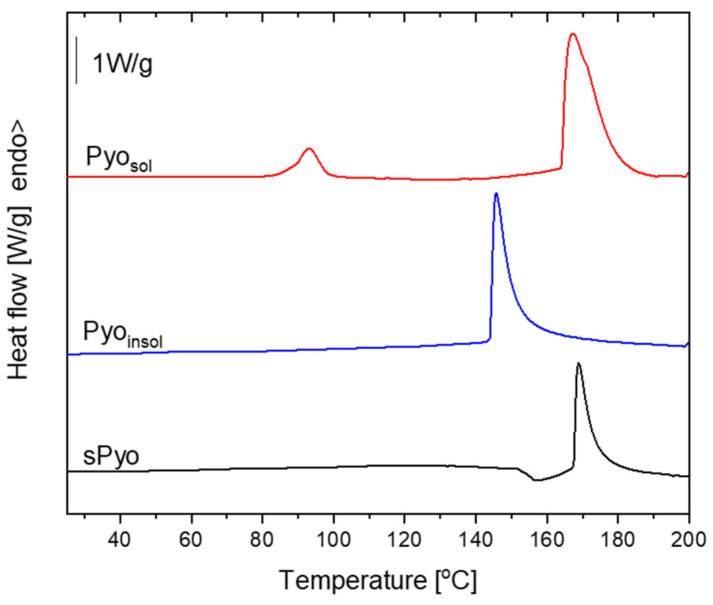
The first heating differential scanning calorimetry (DSC) curves of pyomelanin.

To determine the thermal stability of the pigments, thermogravimetric measurements in an inert atmosphere were performed. Thermal stability is a key parameter determining the possibility of processing pyomelanin by thermal processing methods used for the fabrication of composites and blends with other thermoplastic biopolymers. The TGA curves and the first derivative of the mass with respect to time (dm/dt = f(T)) curves of the dyes are presented in Figure 2, and the estimated characteristic parameters of thermal degradation are collected in Table 2. Based on the shape of the TGA and first derivative curves, it can be concluded that the dyes differed in degradation mechanism. The common feature of the dyes was the presence of the first stage of mass loss occurring up to 125 °C. The maximum rate of the first stage of mass loss, set as the maximum of the first peak on dm/dt = f(T) curves, was located at a similar temperature denoted as the T^1st peak^. This stage of mass loss could be assigned to the loss of volatile low molar mass species. The highest mass loss up to 125 °C was exhibited by Pyo_sol_. Only at a temperature range of mass loss up to 125 °C did the endothermic effect occur for Pyo_sol_, as indicated by the endothermic peak on the DSC curve of Pyo_sol_ (Figure 3). The temperature range of the endothermic effect, the corresponding highest mass loss in the case of Pyo_sol_ and the fact that Pyo_sol_ was isolated from the water phase, allows the assuming of the first stage of mass loss to water loss (Figure 2). The second stage of mass loss could be assigned to the beginning of pyrolysis of pyomelanin. The temperature of onset of the second mass loss can be taken as an upper limit of the thermal stability of pyomelanin (T_deg_^onset^). The highest T_deg_^onset^ was observed for Pyo_insol_ (196.4 °C); for other pyomelanins, T_deg_^onset^ was lower and was located at 173.0 °C and 158.0 °C for Pyo_sol_ and sPyo, respectively. Moreover, for sPyo, above 125 °C, continuous mass loss occurred up to the onset of the main degradation. Thus, Pyo_insol_ and Pyo_sol_ showed superior thermal stability to that of sPyo. Pyo_sol_ had a significantly greater residue at 800 °C (75%) than Pyo_insol_ and sPyo (ca. 40%), indicating a greater proportion of aromatic moieties or other conjugated unsaturated bonds in polymer chains.

The TGA results confirmed that the procedures of isolation and purification of the pigments affect the thermal properties due to differences in the structure and composition of the final products, as indicated by FTIR analysis.

The first heating DSC curves of pyomelanin are presented in Figure 3, and the estimated thermal parameters are detailed in Table 2. On the DSC curves of the pigments, an endothermic peak corresponding to degradation was visible. The localization of the degradation peak maximum (T_deg_^peak^) was similar for sPyo and Pyo_sol_, and that for Pyo_insol_ started at a lower temperature. For Pyo_sol_, a weak endothermic peak with a maximum at 78.5 °C was visible. The assignment of the endothermic effect to water loss agreed with the TGA results. At this temperature range, mass loss occurred for Pyo_sol_, with the highest rate at 85.6 °C, as estimated from the first derivative dm/dt(T) curve. We also observed that Pyo_sol_ exhibited hygroscopic properties.

### 2.4. Biocompatibility

#### 2.4.1. Bacterial Pyomelanins Show Higher Cytocompatibility Than the Synthetic Pyomelanin

**Figure 4:**
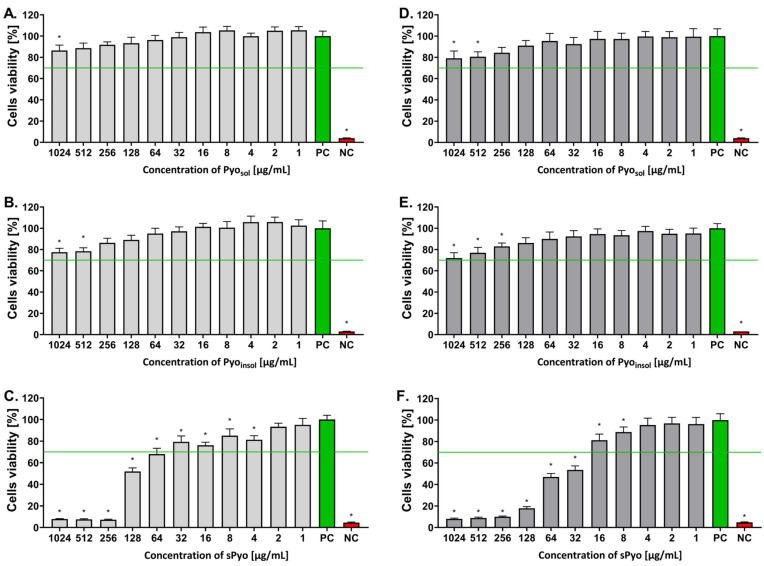
The percentage of viable cells after exposure to different concentrations of the tested pyomelanin variants. Viability of murine fibroblasts L-929 (**A**–**C**) and human monocytes (**D**–**F**) incubated for 24 h with water-soluble pyomelanin (Pyo_sol_) (**A**,**D**), water-insoluble pyomelanin (Pyo_insol_) (**B**,**E**) or synthetic pyomelanin (sPyo) (**C**,**F**), evaluated in the 3-(4,5-dimethylthiazol-2-yl)-2,5-diphenyltetrazolium bromide (MTT) reduction assay according to ISO-10993-5:2009. Cells incubated in the cell culture medium alone, without pyomelanin, served as a positive control (PC) of cell viability (100%). Cells treated with 3% H_2_O_2_ were a negative control (NC) (no viable cells). Data are presented as the mean ± standard deviation (SD) of five separate experiments (six replicates for each experimental variant). The green line indicates the minimum level (70%) of viable cells, which are able to reduce MTT according to the ISO norm. Statistical significance was calculated using ANOVA analysis, followed by Dunnett’s post hoc test. Significant difference *—*p* < 0.05): cells exposed to tested pyomelanins vs. cells in culture medium alone. Statistical significance between Pyo concentrations was calculated using ANOVA analysis, followed by Tukey’s post hoc test. The cytotoxicity of all forms of Pyo was dose-dependent in the concentration range 1024–256 µg/mL (*p* < 0.05).

Looking for new biomedical applications of bacterial polymers, including pyomelanin, requires characterizing their safety at the in vitro level and determining doses that do not show toxic effects. Moreover, differences in structure and thermal stability may translate into interactions of pyomelanin with cells through different ranges of safe concentrations. The influence of each form of pyomelanin on the viability of eukaryotic cells was assessed in the MTT reduction assay using the reference L-929 mouse fibroblasts and human THP-1 monocytes to exclude the cytotoxic effects toward immune cells. The ranges of safe concentrations of different pyomelanin formulations for eukaryotic cells are shown in Figure 4. Pyo_sol_ and Pyo_insol,_ in the full range of tested concentrations (1–1024 µg/mL), did not decrease the number of viable target cells, which were able to reduce MTT (both L-929 and THP-1-cell lines), below the 70% cell level, as required by the ISO norm. The unfavorable effect of reduced cell viability was observed only in the case of sPyo. Less than 70% of mouse fibroblasts or human monocytes were able to reduce MTT in the milieu of sPyo used in the concentration range 64–1024 µg/mL (*p* < 0.05) and 32–1024 µg/mL (*p* < 0.05), respectively (Figure 4).

#### 2.4.2. Water-Soluble Pyomelanin Induces NF-κB Pathway Activation

**Figure 5:**
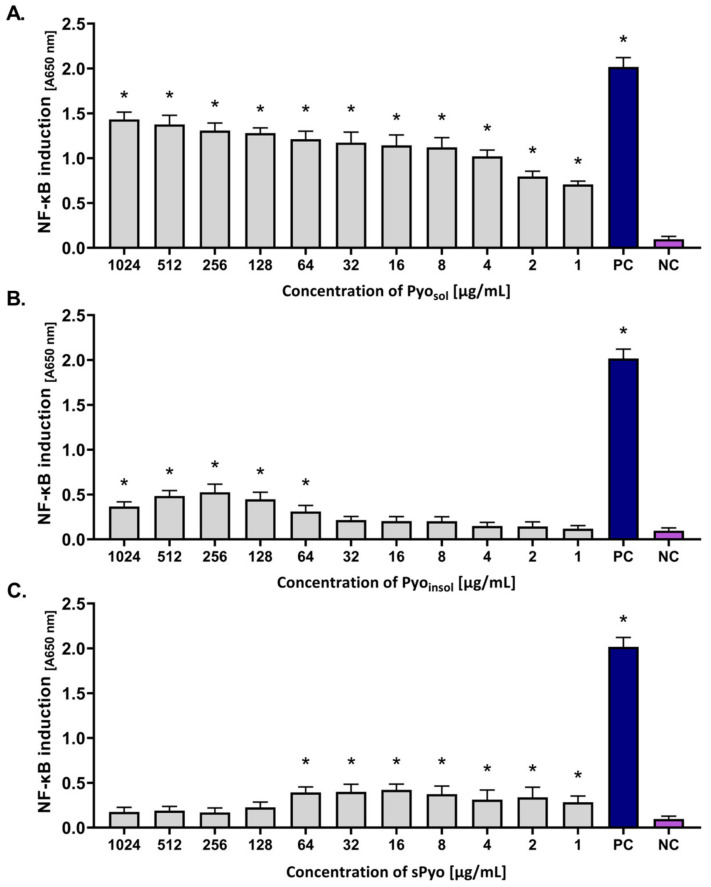
The level of activation of THP1-Blue™ NF-κB monocytes in response to the tested variants of pyomelanin. Cells were incubated for 24 h with (**A**) water-soluble pyomelanin (Pyo_sol_), (**B**) water-insoluble pyomelanin (Pyo_insol_), (**C**) synthetic pyomelanin (sPyo), or lipopolysaccharide (LPS) of *Escherichia coli* as a positive control (PC). Cells in culture medium alone served as the negative control (NC). The secreted embryonic alkaline phosphatase, which was used as an indicator of nuclear factor kappa B (NF-κB) activation, was quantified spectrophotometrically (OD = 650 nm) after enzymatic substrate conversion. Data are presented as the mean ± standard deviation (SD) of five separate experiments (six replicates of each experimental variant). Statistical significance was calculated using ANOVA analysis, followed by Dunnett’s post hoc test. Statistical significance between Pyo concentrations was calculated using ANOVA analysis, followed by Tukey’s post hoc test. Significant difference *, *p* < 0.05. A (absorbance)—optical density 650 nm.

In this study, THP1-Blue™ NF-κB human monocytes were used as biosensors of the NF-κB-driven signaling pathway in innate immune cells. NF-κB induction in these transformed cells results in the SEAP secretion. The amount of SEAP is proportional to cell activation. The levels of induction and activation of NF-κB in THP1-Blue™ NF-κB monocytes in response to the tested variants of pyomelanin are expressed as absorbance in Figure 5.

Pyo_sol_ activated NF-κB in the concentration range of 1–1024 μg/mL (the absorbance ranged from 0.70 to 1.43, *p* < 0.05) (Figure 5A). In cell cultures treated with Pyo_insol_, a significant induction of NF-κB was observed in the concentration range of 64–1024 μg/mL (*p* < 0.05) (Figure 5B). However, the level of activation was lower than that in cell cultures exposed to Pyo_sol_ (absorbance range: 0.30–0.53). Moreover, sPyo induced NF-κB in the concentration range of 1–64 μg/mL; however, there were no differences between induction levels in response to different pyomelanin concentrations within this range (the absorbance range 0.29–0.41) (Figure 5C). The level of NF-κB activation in response to *E. coli* LPS shown as absorbance was equal to 2.07 (*p* < 0.05) (Figure 5A–C).

#### 2.4.3. In Vivo Toxicity of Pyomelanin

**Figure 6:**
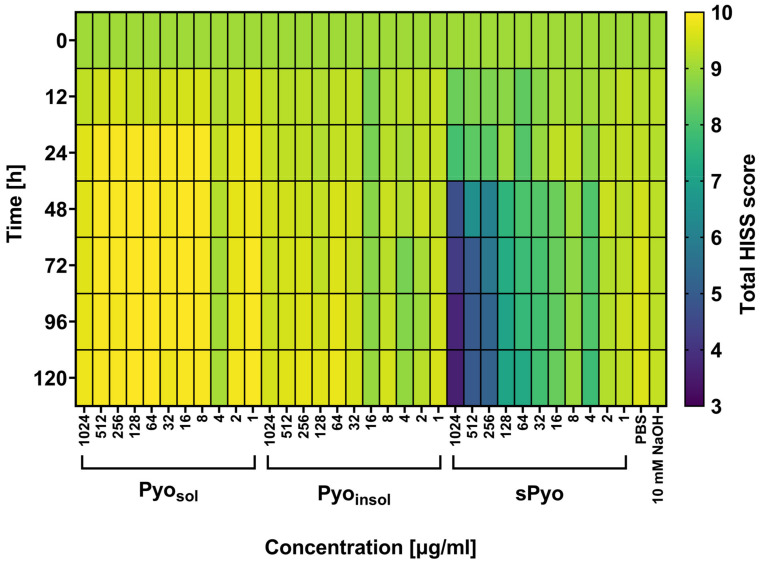
Total Health Index Scoring System (HISS) heatmap for *G. mellonella* larvae treated with tested pyomelanin variants. Larvae were injected with water-soluble (Pyo_sol_), water-insoluble (Pyo_insol_) or synthetic pyomelanin (sPyo) in the concentration range of 1–1024 μg/mL or control solvent solutions: phosphate-buffered saline (PBS) or 50 mM NaOH. At 0, 12, 24, 48, 72, 96, and 120 h after the injection of pyomelanin, the health of the larvae was evaluated using HISS and expressed as the total HISS score. Statistical significance was calculated using ANOVA analysis, followed by Dunnett’s post hoc test. Statistical significance between Pyo concentrations was calculated using ANOVA analysis, followed by Tukey’s post hoc test. sPyo toxicity to *G. mellonella* was dose-dependent in concentration range 1024–64 µg/mL (*p* < 0.05).

In the current study, *G. mellonella* larvae were used as a nonmammalian insect model reflecting the biological complexity of live organisms, which is an ethically accepted alternative for the examination of new formulation safety in vivo. By assessing the four physiological functions of the wax moth larvae after injection with the tested variants of pyomelanin, the HISS of the insects was evaluated and presented as a heatmap (Figure 6). Larvae with a total score in the range of 8.5–10.0 points were regarded as healthy, and the substances tested were considered nontoxic in this in vivo larval model.

As shown in Figure 6, no deleterious effects of the two variants of bacterial pyomelanin (Pyo_sol_ and Pyo_insol_) were seen in the *G. melonella* in vivo model at 0, 12, 24, 48, 72, 96 and 120 h after injection of insect larvae with pyomelanin. The total HISS scores for larvae treated with *P. aeruginosa* pyomelanins were close to the baseline HISS score, which was equal to 9.0. The total HISS scores in larvae injected with sPyo in the range of 4–1024 μg/mL were lower (7.79–3.58) than the total HISS scores in larvae inoculated with natural *P. aeruginosa* pyomelanin variants. Pyo_sol_ and Pyo_insol_ did not affect the viability of insect larvae, while sPyo used in the range of 64–1024 μg/mL significantly decreased the number of live larvae (42–83%, *p* < 0.05) compared to control larvae not injected with pyomelanin (Supplementary Figure S2).

## 3. Discussion

In our study, we described for the first time the method of culturing *Pseudomonas aeruginosa* on the PMM II medium and the method of obtaining water-soluble pyomelanin (Pyo_sol_). In addition, we compared the physicochemical and biological properties of bacterial Pyo_sol_ to its insoluble form (Pyo_insol_) and synthetic pyomelanin (sPyo).

We compared our results with several reports on the efficiency of the production of microbial melanins, which depends on the metabolic abilities of bacteria and culture conditions. Madhusudhan et al. reported a production efficiency of extracellular water-soluble melanin of *Streptomyces lusitanus* at the levels of 0.264 g/L and 5.29 g/L [27]. Lagunas-Muñoz et al. showed that recombinant *E. coli* expressing the tyrosinase coding gene from *Rhizobium* produced 6.0 g/L melanin [28]. The yield of water-insoluble bacterial melanins showed a wide range for different species of bacteria cultured in bioreactors: 13.7 g/L for *Streptomyces kathirae*, 3.76 g/L for *Flavobacterium kingsejongi* and 0.125 g/L for *Streptomyces glaucens* [23,29,30]. Significant differences in melanin production efficiency were also observed within the *Pseudomonas* species, with 6.7 g/L water-insoluble melanin obtained from *Pseudomonas stutzeri* and 0.35 g/L obtained from ***Pseudomonas putida*** [31,32]. A high efficiency of bacterial pyomelanin production with limited secretion of undesirable substances is key to obtaining a bacterial pigment for further physicochemical and biological studies.

The FTIR spectra of microbial Pyo_sol_, Pyo_insol_ and sPyo exhibit the characteristic bands of the pyomelanins described in the literature [33,34]. Based on the relatively high degree of similarity of the spectra of sPyo and Pyo_sol_, it can be concluded that the structure of sPyo is more similar to that of Pyo_sol_ than that of Pyo_insol_. The carbonyl stretching (C=O) of the COOH groups was only visible in Pyo_insol_. The lack of this band can be similarly found in the literature for microbial melanin isolated in acid precipitation [4,35,36]. The FTIR spectrum of Pyo_insol_ was similar to the FTIR spectrum of melanin produced from a deep-sea sponge-associated *Pseudomonas* strain [35] and pyomelanin from a culture of *Halomonas titanicae* which was produced through the 4-hydroxyphenylacetic acid-1-hydroxylase route [6]. Lorquin et al. concluded that the presence of the band ascribed to aromatic C_ar_–H has a significant meaning in terms of the type of ring linkages. This group suggested that a pyomelanin that does not have this band has fewer free C_ar_–H locations and more Car-Car connections between rings in the chain structure [6]. It may suggest that Pyo_sol_ contained more Car-Car linkages than Pyo_insol_. Moreover, the comparison of the FTIR spectra of sPyo and HGA showed that the sPyo did not contain HGA residue detectable by FTIR measurements. Differences in chemical structure may influence the solubility of various forms of pyomelanins, their thermal stability and biological activity; however, further studies are required.

The TGA and DSC results confirmed that the procedures of isolation and purification of the pigments affect the thermal properties due to differences in the structure and composition of the final products, as indicated by FTIR analysis. Pyo_sol_ showed a significantly greater residue at 800 °C than Pyo_insol_ and sPyo, indicating a greater proportion of aromatic moieties or other conjugated unsaturated bonds in polymer chains [37]. This result was in agreement with the FTIR results revealing a higher content of C_ar_–C_ar_ linkages between rings in Pyo_sol_ than in the other pyomelanin samples. For Pyo_sol_, we identified a weak endothermic peak with a maximum at 78.5 °C which is associated with water loss [38]. The presence of this peak only for Pyo_sol_ was a consequence of the isolation of pyomelanin from the aqueous phase. Similar to our DSC results for Pyo_sol_, the showing of two endothermic peaks was reported for microbial melanin by Kiran et al. [35]. We also showed that Pyo_sol_ exhibited hygroscopic properties. Melanin is known for its hygroscopic character and strong association with water [39].

The use of bacterial-derived pyomelanin and synthetic pyomelanin as potential immunomodulators and bioactive substances for further targeted biomedical applications requires determining the range of cytocompatible concentrations to avoid negative effects on cell metabolism and viability. We have shown that bacterial pyomelanins are characterized by high in vitro safety for L-929 fibroblasts and THP-1 monocytes compared to the synthetic form of this pigment. The high level of Pyo_sol_ and Pyo_insol_ cytocompatibility resulted from the effective removal of lipopolysaccharide and other bacterial metabolites that are cytotoxic. The lower cytocompatibility of sPyo compared to Pyosol or Pyo_sol_ against L-929 fibroblasts and human monocytes, as shown in this study, might be due to polymerization and structural differences between sPyo and Pyo_insol_ or Pyo_sol_ (a lower ability to create hydrogen bonds). Interestingly, no residual HGA was observed in the sPyo samples, which could adversely affect cell viability. However, further studies are needed to determine the components influencing the biological activity of the studied pyomelanins. Potentially, the biological activity depends on the complexity of interactions between various functional groups within each variant of pyomelanin.

Several studies have demonstrated the biosafety ranges of microbial melanins in vitro. Oh et al. reported that melanin from *Amorphotheca resinae* in the range of 200–4000 μg/mL did not affect the viability of human keratinocytes HaCaT after 24 h of exposure of cells [40]. Lorquin et al. showed that pyomelanin from *H. titanicae* and its synthetic form resulting from HGA polymerization in the milieu of Mn^2+^ were noncytotoxic to human epidermal keratinocytes [6]. Melanin from *Dietzia schimae*, which possesses photoprotective activity, was safe for human fibroblast hFB at concentrations below ≤500 μg/mL [41]. Pyomelanin produced by various *Pseudomonas* species may differ in cytotoxicity toward eukaryotic cells. In the study by Kurian and Bhat, the highest concentration of melanin from *Pseudomonas stuteri*, which was safe for L-929 fibroblasts, was 100 μg/mL [42]. The cytotoxicity of pyomelanin from *P. putida* against A-375, HeLa Kyoto, HEPG2 or Caco2 cell lines was examined by Ferraz et al. and expressed as the cytotoxicity index IC_50_, with values of 1770 μg/mL, 2510 μg/mL, 890 μg/mL and 1080 μg/mL, respectively [43].

Monocytes play a key role in the development of inflammatory and immune responses, which determine the elimination of infectious agents, induction of antigen-specific adaptive immunity and tissue regeneration; thus, testing new components with medical potential in humans regarding the effectiveness of monocyte activation is needed [44]. The level of activation may vary depending on the cell type, the chemical structure of biocomponents and the cell milieu. In response to tissue damage, monocytes and macrophages deliver proinflammatory cytokines, including chemokines, which facilitate the recruitment of immunocompetent cells, and the removal of debris, which is a prerequisite for successful healing. In subsequent stages of healing, macrophages can reduce inflammation, through the secretion of anti-inflammatory cytokines, can control the differentiation of stem cells, and can regulate angiogenesis [45]. It has been revealed that acute inflammation or low doses of proinflammatory cytokines are necessary for the reconstruction of bone tissue; therefore, modulation of the NF-κB pathway by pyomelanin may influence bone remodeling [46].

In this study, we have shown that Pyo_sol_ is non-cytotoxic in the widest range of concentrations, which is compatible with NF-κB induction compared to the activity of sPyo or Pyo_insol_. However, the poor activation of NF-κB by water-insoluble and synthetic forms of pyomelanin does not rule them out from further biological studies. Depending on the application context, the proinflammatory or anti-inflammatory activity of pyomelanin can be considered. Proinflammatory activity is desirable in fighting against infection and in the early stages of tissue regeneration, whereas to prevent chronic inflammation, anti-inflammatory properties of biocomponents are needed. Allam et al. reported that *Streptomyces longisporoflavus* melanin improves immune defense against *Escherichia coli* infection [47]. The severity of oxidative stress is the result of a strong inflammatory response due to the activation of monocytes. Langhfelder et al. reported on the ability of fungal melanins to neutralize such stress [11]. On the other hand, the initiation of regeneration processes requires the stimulation of monocytes to secrete proinflammatory cytokines, which was recently reported [19]. The Pyo_sol_ used in this study seems to meet the highest biological safety, combined with the activation of monocytes, and due to this may be further tested for immunomodulatory and pro-regenerative properties in different cell models.

The results from the in vivo model of *G. mellonella* obtained in this study correspond to the observations on the safety of various forms of pyomelanin in the in vitro model. These results are very useful in selecting potential pyomelanin applications. In particular, Pyo_sol_ and Pyo_insol_ seem to meet the requirements for further studies on potential medical applications. In contrast, sPyo can be further investigated for nonmedical applications.

## 4. Materials and Methods

### 4.1. Culture of Pseudomonas aeruginosa

To obtain pyomelanin with a limited content of undesirable substances (e.g., alginate, excess protein products), a new minimal liquid medium for *P. aeruginosa* cultivation was developed. Two versions of pyomelanin minimal medium (PMM) were prepared. The first PMM version (PMM I) contained 2.0 g of KH_2_PO_4_, 5.0 g of NaCl, 0.1 g of MgSO_4_, 2.0 g of L-tyrosine and 2.0 g of glucose per 1000 mL of distilled water. The second version (PMM II) was additionally supplemented with 1.5 g of arabinose and 1.35 g of malic acid. All chemicals were purchased from PolAura, Dywity, Poland. After dissolving the substrates, the pH of both media was adjusted to 7.0 with 0.5 M NaOH. PMM I and PMM II were autoclaved at 121 °C, 2.5 Ba. Luria–Bertani (LB) broth medium was inoculated with the *P. aeruginosa* Mel^+^ strain deposited in the collection of the Department of Immunology and Infectious Biology UŁ, Poland, and cultured (37 °C, 18 h) to obtain an initial bacterial suspension. After incubation, 300 mL of PMM II was inoculated with 1.0 mL of a 1.0 McFarland bacterial suspension and grown for 5 days (37 °C, shaking at 120 rpm) when the culture medium changed to a deep black-brown color. To increase the production of pyomelanin, after cultivation, the bacterial cultures were exposed to sunlight for 2 days at room temperature.

### 4.2. Isolation of Pyo_insol_

To isolate Pyo_insol_ from the cell-free supernatant, 300 mL of bacterial culture was centrifuged at 3300× *g* for 15 min, and then the supernatant was acidified with 6.0 M HCl (PolAura, Dywity, Poland) to pH 2.0 and stored, protected from light, at room temperature for 5 days. Thereafter, the supernatant was boiled for 45 min to avoid the formation of melanoidins after cooling, and the supernatant was centrifuged at 3300× *g* for 25 min. The pellet of Pyo_insol_ was washed three times with 25 mL of 0.1 M HCl and then three times with double distilled water. Afterward, 10 mL of ethanol (99.9%) (Chempur, Piekary Śląskie, Poland) was added to the pigment pellet and placed in a water bath (95 °C, 30 min). After storage in an incubator (50 °C, overnight), for complete evaporation of alcohol, the pyomelanin was washed twice with ethanol and air dried.

### 4.3. Isolation of Pyo_sol_

To obtain Pyo_sol_, the postculture bacterial cell-free supernatant was incubated with chloroform in a 1:1 ratio under shaking conditions for 24 h (room temperature, shaking at 120 rpm). The aqueous phase containing pyomelanin was then separated from the chloroform and protein phases using a separating funnel. To remove residual protein contaminants, the aqueous layer was centrifuged at 5300× *g* for 30 min. Pyo_sol_ was concentrated and purified from low-molecular-weight soluble substances by centrifuging the supernatant on an ultrafiltration unit (3300× *g*, 60 min., MWCO 30 kDa) (Sartorius, Göttingen, Germany). The bacterial pigment was dried at 50 °C overnight.

### 4.4. Synthesis of Pyomelanin

HGA (TCI, Eschborn, Germany), which is the main precursor for pyomelanin in *P. aeruginosa*, was used to prepare sPyo. HGA (1.0 g) was dissolved in 400 mL of distilled water (heated to 50 °C), and then a solution of 4.0 M NaOH was added to achieve pH 10.5. Autoxidation of HGA to sPyo was carried out for 10 days at 37 °C in the absence of light. The tube with the HGA solution was opened once a day to provide a new portion of oxygen. When a dark brown pigment was observed in the tube, sPyo was precipitated with 10.0 M HCl (to pH 6.0), left to sediment for 24 h and centrifuged (3300× *g*, 25 min). The pyomelanin pellet was suspended in 2.2 M HCl and left for 2 days to stabilize the pigment. Then, sPyo was centrifuged (6600× *g*, 10 min) and washed three times with 0.1 M HCl and double-distilled water.

### 4.5. Purification of Bacterial Pyomelanins

**Figure 7:**
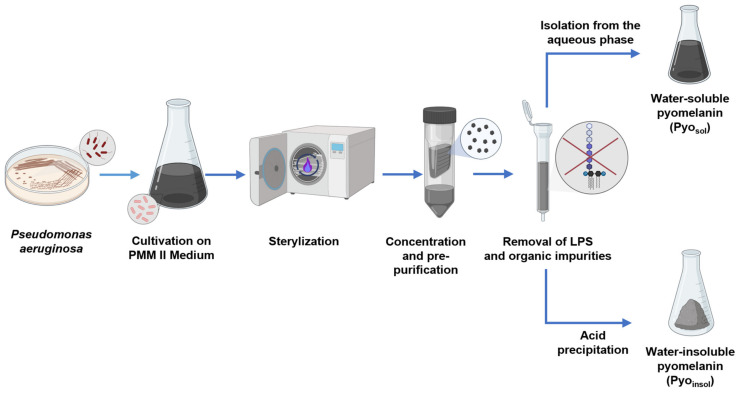
Schematic representation of isolation and purification of bacterial pyomelanins. Abbreviations: water-soluble pyomelanin (Pyo_sol_), water-insoluble pyomelanin (Pyo_insol_), synthetic pyomelanin (sPyo), lipopolysaccharide (LPS).

Lipopolysaccharides (LPSs) were removed from *P. aeruginosa* pyomelanin by affinity chromatography using Pierce™ High Capacity Endotoxin Removal Spin Columns (Thermo Scientific, Waltham, MA, USA). The resin and column were prepared and equilibrated according to the manufacturer’s protocol. Samples of Pyo_sol_ and Pyo_insol_ (5 mg/mL) were applied to the columns, incubated for 3 h with gentle mixing, centrifuged at 500× *g*, collected into new tubes and dried at 50 °C overnight. The pyomelanin pellet was washed with chloroform, ethyl acetate, ethanol and water. For further experiments, the pyomelanin was stored in a dark and dry place at 4 °C. The methodology for the isolation and purification of bacterial pyomelanins is shown in Figure 7.

### 4.6. Fourier Transform infrared (FTIR) Spectroscopy

FTIR spectra in transmission mode were collected from 4000 to 400 cm^−1^ using the KBr pellet technique on a Thermo Nicolet Nexus FTIR spectrometer (Thermo Fisher Scientific, Waltham, MA, USA) and analyzed with Thermo Scientific Omnic Software ver. 8.3.

### 4.7. Thermogravimetric Analysis (TGA)

TGA measurements were performed using a TGA/DSC1 Mettler Toledo system (Mettler Toledo, Greifenesee, Switzerland) [48]. Samples were heated from 25 °C to 800 °C at a rate of 10 °C/min under 60 mL/min of nitrogen flow. The evaluation of the TGA curves was performed using STARe ver. 16.20c software (Mettler Toledo, Greifenesee, Switzerland). The first derivative of mass over time was calculated with OriginPro ver. 2021 (OriginLab Corporation, Northampton, MA, USA) and plotted against temperature. The Savitzky-Golay smoothing algorithm was implemented. A 20 point window and a second-order polynomial was used.

### 4.8. Differential Scanning Calorimetry (DSC)

DSC measurements were performed using a Mettler Toledo DSC1 system (Mettler Toledo, Greifenesee, Switzerland) coupled with a Huber TC 100 intracooler (Huber USA, Inc., Raleigh, USA) [49]. The instrument was calibrated using indium (T_m_ = 156.6 °C, ΔH_m_ = 28.45 J/g) and zinc (T_m_= 419.7 °C, ΔH_m_ = 107.00 J/g) standards. Samples (~3.5 mg) were measured in 40 μL aluminum pans under a constant nitrogen purge (60 mL/min) from 0 °C to 200 °C. The heating and cooling rates were set to 10 °C/min. The recorded DSC curves were normalized to the sample mass. The evaluation of the DSC curves was performed using STAR^e^ ver. 16.20c software (Mettler Toledo, Greifenesee, Switzerland).

### 4.9. Assessment of Pyomelanin Biocompatibility

#### 4.9.1. Cell Cultures

The biocompatibility of pyomelanin was assessed in vitro according to ISO 10993-5:2009 (Biological evaluation of medical devices—Part 5: Tests for in vitro cytotoxicity) using two cell lines: the reference L-929 (CCL-1™) mouse fibroblasts and human monocytes THP-1 (TIB-202™), which were obtained from the American Type Culture Collection (ATCC, Manassas, VA, USA). Prior to experiments, cells were cultured in Roswell Park Memorial Institute (RPMI)-1640 medium supplemented with 10% heat-inactivated fetal calf serum (FCS; HyClone Cytiva, Marlborough, MA, USA) and the antibiotics penicillin (100 U/mL) and streptomycin (100 µg/mL) (Sigma-Aldrich, Darmstadt, Germany). Mouse fibroblasts and human monocytes were incubated at 37 °C in a humidified atmosphere containing 5% CO_2_ until the formation of the cell monolayer. Before being used in the experiments, the cell viability and cell density were assessed by trypan blue exclusion assay using a counting Bürker chamber (Blaubrand, Wertheim, Germany). The cells were used in the experiments only if cell viability was higher than 95%.

#### 4.9.2. MTT Reduction Assay

The biocompatibility of pyomelanins was assessed in vitro in cell cultures using a 3-(4,5-dimethylthiazol-2-yl)-2,5-diphenyltetrazolium bromide (MTT, Sigma-Aldrich, Darmstadt, Germany) reduction assay as previously described [50] and as recommended by the Food and Drug Administration and ISO norm 109935 [ISO 10993-10995:2009. Biological evaluation of medical devices—Part 5: Tests for in vitro cytotoxicity]. L-929 fibroblasts or THP-1 monocytes adjusted to a density of 2 × 10^5^ cells/mL were seeded (20,000 cells per well) in 96 well culture plates (Nunclon Delta Surface, Nunc, Rochester, NY, USA) and incubated overnight prior to stimulation with pyomelanin. Cell morphology and confluency were controlled using an inverted contrast phase microscope (Motic AE2000, Xiamen, China). Stock solutions of Pyo_insol_ or sPyo, 10 mg/mL in 50 mM NaOH (PolAura, Dywity, Poland) in complete RPMI-1640 (cRPMI-1640) were diluted with medium to concentrations of 1024, 512, 256, 128, 64, 32, 16, 8, 4, 2 and 1 μg/mL. An identical series of dilutions was prepared for Pyo_sol_ starting from a stock solution at a concentration of 2 mg/mL initially dissolved in cRPMI-1640. The pyomelanin solutions were sterilized by filtration using filters with a 0.22 μm pore diameter (Sartorius, Göttingen, Germany). Suspensions of pyomelanins were distributed to the wells of culture plates (6 replicates for each experimental variant) containing cell monolayers. After 24 h of incubation, the condition of the cell monolayers was verified under an inverted contrast phase microscope. The cell cultures in medium without pyomelanin were used as a positive control (PC) of cell metabolic activity, whereas the cell cultures in 3% H_2_O_2_ served as a negative control (NC). To quantify the metabolic activity of cells, 20 µL of MTT was added to each well, and incubation was carried out for the next 4 h. The plates were centrifuged at 450× *g* for 10 min, the supernatant was removed, and the formazan crystals were dissolved with 100 µL of dimethyl sulfoxide (Sigma Aldrich, Seelze, Germany). The absorbance was determined spectrophotometrically using a Multiskan EX reader (Thermo Scientific, Waltham, MA, USA) at 570 nm. The effectiveness of MTT reduction was calculated based on the following formula: MTT reduction relative to untreated cells (%) = (absorbance of treated cells/absorbance of untreated cells × 100%) − 100%.

#### 4.9.3. Activation of Monocytes

THP1-Blue™ NF-κB monocytes (InvivoGen, San Diego, CA, USA), derived from human THP-1 monocytes, were used to determine the activation of the NF-κB signal transduction pathway, as previously described [51], in response to exposure of cells to sPyo, Pyo_insol_ or Pyo_sol_. THP1-Blue™ NF-κB cells are specific biosensors of the NF-κB pathway, which is typical for innate immune cells [52,53]. The induction of NF-κB results in secretion of embryonic alkaline phosphatase (SEAP) by these cells. Cell suspensions, 2 × 10^6^ cells/mL in selective RPMI-1640 supplemented with heat-inactivated 10% FCS (HyClone, Cytiva, Marlborough, MA, USA), 25 mM 4-(2-hydroxyethyl)-1-piperazineethanesulfonic acid (HEPES), 100 U/mL penicillin, 100 µg/mL streptomycin, 2 mM glutamine and selective antibiotics (100 µg/mL normocin and 10 µg/mL blasticidin) (InvivoGen, San Diego, CA, USA), at a density below 2 × 10^6^ cells/mL, were cultured for 5 days in a humidified 5% CO_2_ atmosphere at 37 °C. Freshly prepared suspensions of monocytes in culture medium were distributed to the wells of culture plates (1 × 10^5^ cells/well; 180 µL). Then, 20 µL of tenfold-concentrated pyomelanin solution was added to selected wells (in six replicates) to a final concentration of 1024, 512, 256, 128, 64, 32, 16, 8, 4, 2 and 1 μg/mL. Cells were incubated for 24 h in an incubator. Monocytes in selective RPMI-1640 alone served as an negative control (NC), whereas monocytes stimulated with 10 ng/mL LPS from *Escherichia coli* O55:B5 (Sigma-Aldrich, Darmstadt, Germany) were used as a positive control (PC) for NF-κB activation. The level of SEAP secretion was determined in the cell culture supernatants. Cell-free supernatant (20 µL) was mixed with 180 µL QUANTI-Blue™ (InvivoGen, San Diego, CA, USA) and incubated at 37 °C for 4 h. Absorbance was measured at 650 nm using a Multiskan EX reader (Thermo Scientific, Waltham, MA, USA). The results are expressed as the mean and standard deviation (SD) of five experiments performed in six replicates for each experimental variant.

#### 4.9.4. In Vivo Toxicity Assay

Different formulations of pyomelanin were examined for their toxicity in vivo using the model of last instar *Galleria mellonella* larvae, which enables real-time cytotoxicity testing [53,54,55]. In the assessment of the last instar, the size of the larva, width and degree of scleritization of the head capsule were analyzed, and the ecdysial line along the middle of the dorsal side was observed. Before the experiment, the last instar of larvae (purchased from a local vendor), confirmed by the specialist from the Department of Ecology and Vertebrate Zoology, Faculty of Biology and Environmental Protection, University of Łódź, Poland, were stored in the dark in a refrigerator at 15 °C to minimize transformation into adult form. Prior to conducting the assay, each larva was sterilized with a cotton swab dipped in 70% ethanol. The bioassay was performed in glass Petri dishes. Twelve larvae (200–300 mg weight) were injected with 10 μL of Pyo_insol_, Pyo_sol_ or sPyo into the hemocoel via the intersegmental membrane near the last left proleg using a microsyringe (Sigma Aldrich, Darmstadt, Germany). Control larvae were injected with phosphate-buffered saline (PBS) or 10 mM NaOH (control solvent for Pyo_sol_ and sPyo). The physiological activities of insects or the number of dead insects were recorded at 0, 12, 24, 48, 72, 96 and 120 h after the injection. The larval health status was evaluated using the Health Index Scoring System (HISS), which is based on the following symptoms: larval mobility, cocoon formation, melanization of the body integuments and survival [55] (Table 3). In this system, the total melanization of the larvae (black larvae) and loss of larval motility correlate with the death of the larvae [56]. Representative pictures of morphological changes in larvae injected with Pyo_sol_, Pyo_insol_ or sPyo are shown in Supplementary Figure S1.

### 4.10. Statistical Analysis

The Kolmogorov–Smirnov test was used to test the normality of the data. Intergroup outcomes were compared for statistical significance using ANOVA (analysis of variance) followed by Dunnett’s post hoc test. Statistical significance between pyomelanin concentrations was calculated using ANOVA analysis, followed by Tukey’s post hoc test. In all cases, significance was accepted at *p* < 0.05. All analyses were performed using GraphPad Prism 9 software (GraphPad Software, San Diego, CA, USA).

## 5. Conclusions

Taking into account the need to search for biocomponents, including those of bacterial origin with multidirectional biological activity (antimicrobial, immunomodulatory and proregenerative), the aim of this study was to obtain natural bacterial pyomelanin from *Pseudomonas aeruginosa*, namely, Pyo_sol_ and Pyo_insol_, and synthetic pyomelanin (sPyo). Furthermore, we characterized these three variants of pyomelanin in terms of chemical structure and biosafety for further targeted biomedical research. The culture medium increasing the production of pyomelanin was developed as well as the conditions for the isolation and synthesis of pyomelanin from HGA. FTIR analysis showed that the most important difference between variants of pyomelanin concerns the connections of aromatic rings in polymer chains. In the case of Pyo_sol_ and sPyo, the C_ar_-C_ar_ connections between rings dominate the chain structure, whereas Pyo_insol_ showed fewer C_ar_-C_ar_ links between rings. For Pyo_sol_, the wide band at 3700–3000 cm^−1^ indicated the presence of condensed double bonds as well as the presence of OH groups involved in hydrogen interactions, e.g., with water. We observed that Pyo_sol_ exhibited hygroscopic properties (atmospheric moisture caused clumping). TGA confirmed the highest water content in Pyo_sol_. Further chemical research is required to fully define the structural differences in the molecules of different variants of pyomelanin. A high level of biological safety allows for the recommendation of Pyo_sol_ and Pyo_insol_ for further biomedical studies, including research on their antimicrobial, immunomodulatory, and proregenerative activities. Investigating the biosafety and modulation of the physiological activity of other cell lines will guide research on pyomelanin towards the development of pyomelanin bioactive preparations.

## Figures and Tables

**Table 1:** Efficiency of the Extraction of *P. aeruginosa* Pyomelanin from Minimal Growth Media and the Synthesis of Pyomelanin from HGA.

*P. aeruginosa* Growth Medium	Pyo_sol_ [g/L]	Pyo_insol_ [g/L]	sPyo [g/1 g of HGA]
PMM I	1.13 ± 0.12	0.71 ± 0.04	0.077 ± 0.01
PMM II	1.79 ± 0.18	1.22 ± 0.10	

**Table 2:** Thermal Stability Parameters of the Polymers Estimated from Thermogravimetry and Thermal Properties from Differential Scanning Calorimetry.

Form of Pyomelanin	T^1st peak^ [°C]	Mass Loss Up to 125 °C [%]	T_deg_^onset^ [°C]	T_deg_^peak^ [°C]	Residue at 800 °C [%]	T_1_ [°C]	ΔH_1_ [J/g]
sPyo	64.2	2.90	158.0	176.3	39.55	—	—
Pyo_insol_	72.0	1.60	196.4	233.3	38.85	—	—
Pyo_sol_	85.6	7.09	173.0	193.7	75.91	78.5	19.8

**Table 3:** The Health Index Scoring System (HISS) for *G. mellonella* Larvae.

Category	Description	Score
Mobility	no movement	0
	minimal movement on stimulation	1
	movement when stimulated	2
	movement without stimulation	3
Cocoon formation	no cocoon	0
	partial cocoon	0.5
	full cocoon	1
Melanization	black larvae	0
	black spots on brown larvae	1
	≥3 spots on beige larvae	2
	<3 spots on beige larvae	3
	no melanization	4
Survival	dead	0
	alive	2

## Data Availability

The data generated during this study are available at University of Łódź, Faculty of Biology and Environmental Protection, Department of Immunology and Infectious Biology, Łódź, 90-237, Poland, and are available from the corresponding authors upon request.

## Supplementary Materials

The following supporting information can be downloaded at: https://www.mdpi.com/article/10.3390/ijms24097846/s1.
