# Supplementary material for: In Vitro and In Vivo Biocompatibility of Natural and Synthetic Pseudomonas aeruginosa Pyomelanin for Potential Biomedical Applications
Source: Int J Mol Sci. 2023 Apr 25;24(9):7846. doi: 10.3390/ijms24097846 (PMC10178424; doi:10.3390/ijms24097846)
Supplement: Supplementary file 1 [file ijms-24-07846-s001.zip › ijms-2213203-supplementary.pdf]

In Vitro and In Vivo Biocompatibility of Natural and Synthetic *Pseudomonas aeruginosa* Pyomelanin for Potential Biomedical Applications

Mateusz M. Urbaniak [1,2], Małgorzata Gazińska [3], Karolina Rudnicka [1], Przemysław Płociński [1], Monika Nowak [1] and Magdalena Chmiela [1,*]

[1] Department of Immunology and Infectious Biology, Faculty of Biology and Environmental Protection, University of Łódź, 90-237 Łódź, Poland

[2] The Bio-Med-Chem Doctoral School, University of Lodz and Lodz Institutes of the Polish Academy of Sciences, 90-237 Łódź, Poland

[3] Department of Engineering and Technology of Polymers, Faculty of Chemistry, Wrocław University of Science and Technology (WUST), 50-370 Wrocław, Poland

SUPPLEMENTARY MATERIAL

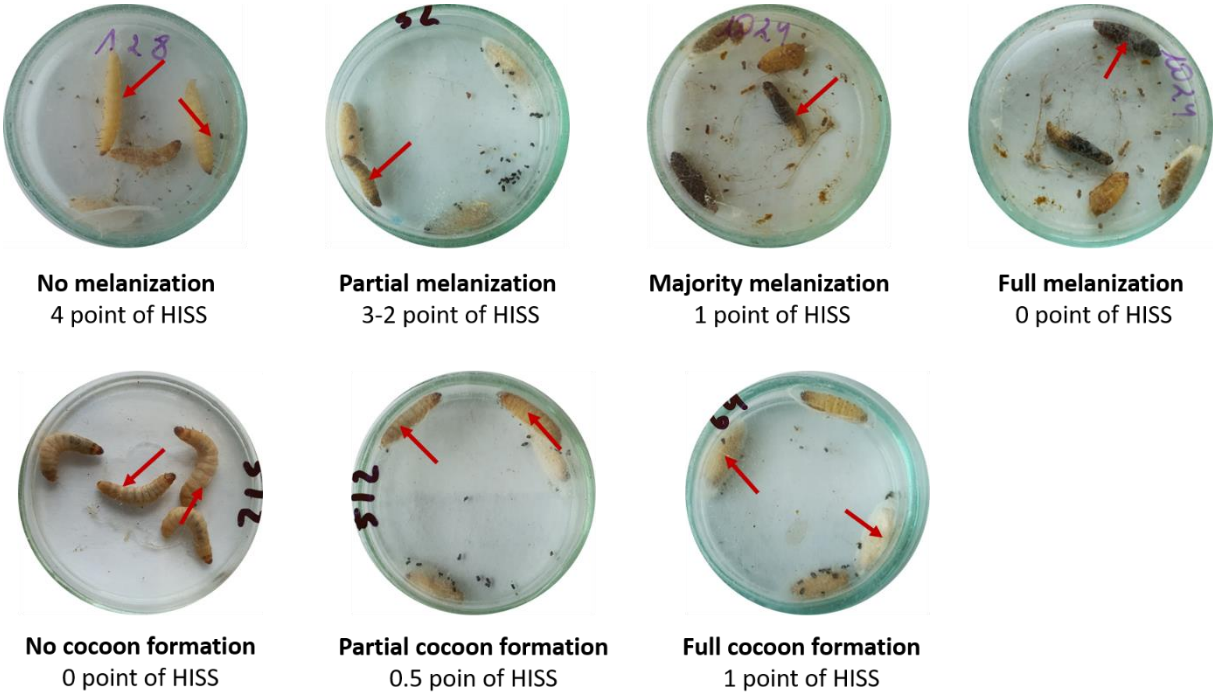

Supplementary Figure S1. Larval morphological changes observed in melanization and cocoon formation during Health Index Scoring System (HISS) assessment.

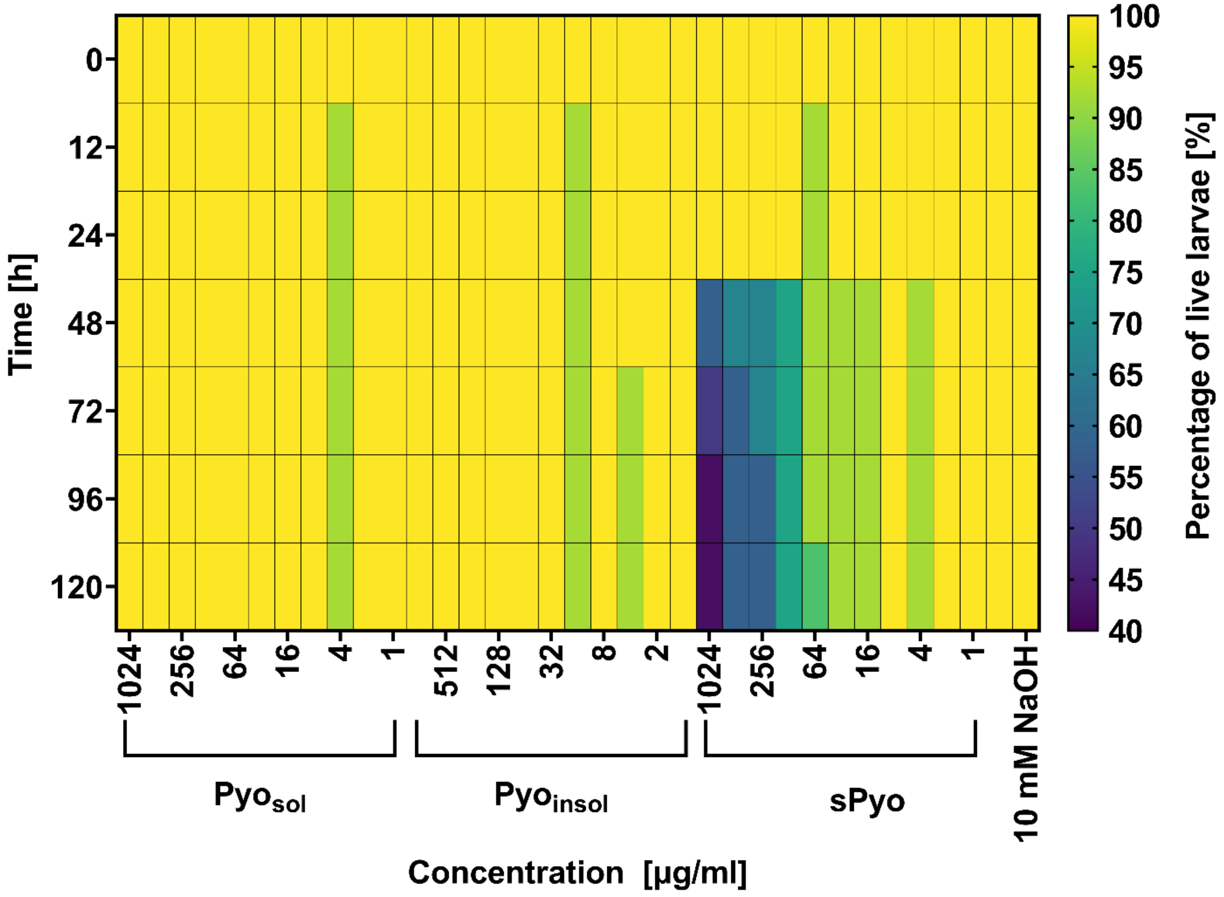

Supplementary Figure S2. Heatmap of toxicity for *G. mellonella* at 0, 12, 24, 48, 72, 96, and 120 hrs from the injection of water-soluble (Pyo$_{sol}$), water-insoluble (Pyo$_{insol}$), and synthetic (sPyo) pyomelanin in the concentration range 1-1024 µg/mL and control substances: phosphate buffer (PBS) and 50 mM NaOH.
